# Genome Analysis of a Highly Virulent Serotype 1 Strain of *Streptococcus pneumoniae* from West Africa

**DOI:** 10.1371/journal.pone.0026742

**Published:** 2012-10-17

**Authors:** Tiffany M. Williams, Nicholas J. Loman, Chinelo Ebruke, Daniel M. Musher, Richard A. Adegbola, Mark J. Pallen, George M. Weinstock, Martin Antonio

**Affiliations:** 1 The Genome Institute, Washington University, St. Louis, Missouri, United States of America; 2 Centre for Systems Biology, University of Birmingham, Birmingham, United Kingdom; 3 Bacterial Diseases Programme, Medical Research Council Laboratories, Banjul, The Gambia; 4 Infectious Diseases Section, Michael E. DeBakey VA Medical Center, Houston, Texas, United States of America; Charité-University Medicine Berlin, Germany

## Abstract

*Streptococcus pneumoniae* is a leading cause of pneumonia, meningitis, and bacteremia, estimated to cause 2 million deaths annually. The majority of pneumococcal mortality occurs in developing countries, with serotype 1 a leading cause in these areas. To begin to better understand the larger impact that serotype 1 strains have in developing countries, we characterized virulence and genetic content of PNI0373, a serotype 1 strain from a diseased patient in The Gambia. PNI0373 and another African serotype 1 strain showed high virulence in a mouse intraperitoneal challenge model, with 20% survival at a dose of 1 cfu. The PNI0373 genome sequence was similar in structure to other pneumococci, with the exception of a 100 kb inversion. PNI0373 showed only15 lineage specific CDS when compared to the pan-genome of pneumococcus. However analysis of non-core orthologs of pneumococcal genomes, showed serotype 1 strains to be closely related. Three regions were found to be serotype 1 associated and likely products of horizontal gene transfer. A detailed inventory of known virulence factors showed that some functions associated with colonization were absent, consistent with the observation that carriage of this highly virulent serotype is unusual. The African serotype 1 strains thus appear to be closely related to each other and different from other pneumococci despite similar genetic content.

## Introduction

Globally, *Streptococcus pneumoniae* is a leading cause of pneumonia, meningitis, and bacteremia, collectively termed invasive pneumococcal disease (IPD) [1], [2]. It is estimated that *S. pneumoniae* is responsible for 2 million deaths annually, with 0.7–1 million of these occurring in children < five years of age [3]–[5]. The case fatality rate for IPD ranges from 11% for pneumonia among adults within industrialized nations to >50% for meningitis within children <5 years of age in sub-Saharan Africa, the majority of pneumococcal mortality occurring in developing countries [6]–[8].

In many of high pneumococcal burden countries within Africa, Asia and Latin America, serotype 1 consistently ranks among the most prevalent IPD-causing serotypes in children and adults [9]–[13]. Despite limited nasopharyngeal colonization and low levels of antibiotic resistance, this serotype behaves as a primary pathogen, in contrast to other serotypes, and is frequently associated with disease outbreaks [14]–[20]. Furthermore within the African meningitis belt, characterized by its high incidence of meningitis and associated mortality, almost 60% of pneumococcal meningitis is attributable to serotype 1 strains [21]. Additionally, no evidence of protection against serotype 1 was apparent in Gambian and South African trials evaluating an expanded pneumococcal glycoconjugate vaccine, which included the serotype 1 glycoprotein, although the total serotype 1 cases were small and thus statistical power was insufficient to draw conclusions [22], [23].

Molecular epidemiology of global serotype 1 reveals a geographically-structured clonal population [23]–[25]. The limited genetic diversity is likely attributable to short carriage duration and/or low bacterial density during colonization which act in concert to reduce opportunity for genetic exchange [25], [26]. Based on data from the *S. pneumoniae* MLST database (http://spneumoniae.mlst.net/) and other surveillance studies, the predominant serotype 1 genotypes in Africa are highly related with most belonging to the same clonal complex, CC217, which includes ST217, ST618, ST303 and ST612 among others [15], [19]; a recent expansion of a hypervirulent ST618 has been noted in The Gambia [23].

Given the relative paucity of genomic data from non-Western pneumococcal isolates, and the growing body of evidence emphasizing the pathogenicity of serotype 1 in Africa and its potential implications for vaccine efficacy, we sought to evaluate the virulence in mice and describe the genetic features of a clinically relevant prototypic serotype 1 isolate from Africa. Strain PNI0373 is a ST618 serotype 1 isolate recovered from a blood sample taken from a pediatric patient with a lethal case of pneumococcal bacteremia in The Gambia, year 2000. As is characteristic for serotype 1, antibiotic resistance was not detected in this strain.

## Materials and Methods

### Ethics statement

All animal experimentation was carried out in accordance with institutional guidelines and following Institutional Review Board approval. The work described in this report was approved by the Baylor College of Medicine Institutional Review Board under protocols AN-3326 (approved January 19, 2005 through January 20, 2007) and AN-4885, (approved May 9, 2008 through March 25, 2011).

### Bacterial strains and animal studies

Strains used in animal experiments include TIGR4 (BAA-334), R6 (BAA-255), D39, P1031 and PNI0373. The former three were obtained from ATCC while D39 and P1031 were graciously provided to us by Daniel M. Musher, M.D. and Vega Masignani, Ph.D. at Novartis Vaccines in Siena, Italy, respectively (Table 1). All strains were chosen because each had genomic data available and each was either (1) a serotype 1 isolate or (2) a well-studied isolate used in previous reports of murine IPD models. Use of well-studied pneumococcal strains allowed us to establish a relative level of virulence for PNI0373 and P1031 compared to that of TIGR4, D39 and R6.

**Table 1:** Overview of general genomes features of 12 complete pneumococcal genomes.

	PNI0373	P1031	D39	R6	TIGR4	70585	CGSP14	JJA	Hungary 19A-6	G54	Taiwan 19F-14	ATCC 700669
Serotype	1	1	2	NT	4	5	14	14	19A	19F	19F	23F
MLST ST	ST618	ST303	ST128	ST128	ST205	ST289	ST15	ST66	ST168	ST63	ST236	ST2
Location of isolation	The Gambia	Ghana	USA	USA	Norway	Bangladesh	Taiwan	Brazil	Hungary	Italy	Taiwan	Spain
Accession #	CP001845	CP000920	CP000410	AE007317	AE005672	CP000918	CP001033	CP000919	CP000936	CP001015	CP000921	FM211187
Genome size (bp)	2064154	2111882	2046115	2038615	2160842	2184682	2209198	2120234	2245615	2078953	2112148	2221315
% G+C	39.81	39.75	39.71	39.71	39.7	39.73	39.46	39.74	39.63	39.64	39.77	39.49
% coding	85	83	83	86	83	84	86	84	82	85	82	82
Predicted proteins	2121	2073	1914	2042	2105	2202	2206	2123	2155	2115	2044	1990
Pseudogenes	31	104	82	N/A	126	44	N/A	42	177	N/A	84	141
Structual RNAs	75	77	73	73	70	77	70	70	70	71	77	90
tRNAs	57	58	58	58	58	58	58	58	55	58	58	58
rRNAs	12	12	12	12	12	12	12	12	12	12	12	12
Other RNAs	6	7	3	3	0	7	0	0	3	1	7	20

Outbred 7-week old female CD-1 mice were obtained from Charles River Laboratories. We elected to use a well-studied outbred line to recapitulate the variation in susceptibility to disease seen in non-inbreeding human populations. Animals were allowed to acclimate to their new environment for 4–7 days prior to initiation of experiments. Mice were caged in groups of five in standard housing and given a standard diet. All animal experimentation was carried out in accordance to institutional guidelines following Institutional Review Board approval.

Bacteria were grown from freezer stocks by streaking them onto blood agar plates (TSA with 5% sheep blood, Remel, Lenexa, KS, USA) and allowed to grow for 16–20 hours at 37°C with 5% CO_2_. Bacteria were then resuspended in 1 ml of chilled 5% sterile saline. Serial dilutions of saline resuspensions were prepared and kept on ice prior to murine challenge. To study post-invasive virulence, groups of five non-anesthetized mice were given 100 µl final volume intraperitoneal injections of 10^6^ cfu bacteria. Mice were closely monitored every four hours for seven days post-infection. When mice were observed to be moribund, defined as dehydration, ruffled fur, hunched posture, poor mobility, pallor, and/or respiratory distress, they were sacrificed. Mortality data were collected and recorded.

The level of bacteremia in mice challenged with PNI0373 was assessed as follows. Bacteria were prepared for challenge in the same manner as for post-invasive experiments. A total of 40 mice were inoculated with 100 µl final volume of 10^6^ cfu bacteria intraperitoneally. At four-hour time points, blood samples were recovered from groups of five mice by exsanguination. Total bacterial burden per ml of blood were determined by enumerating colony forming units from serial dilutions of blood samples.

For median lethal dose (LD50) determination of PNI0373, bacteria were grown as outlined above. Ten-fold dilutions beginning at 10^6^ cfu and proceeding to 10^0^ cfu were prepared in 0.9% chilled saline. Groups of five mice received 100 µl intraperitoneal injections of bacterial log-fold dilutions. Mice were again monitored for the seven days post-infection and sacrificed when determined to be moribund. Survival data were recorded.

### Bacterial DNA preparation

*S. pneumoniae* strain PNI0373 was obtained from the Medical Research Council Laboratories, The Gambia, as a freezer stock from a single colony culture. This freezer stock was streaked on blood agar plates (TSA with 5% sheep blood, Remel, Lenexa, KS, USA) and allowed to grow for 16–20 hours at 37°C with 5% CO_2_. Genomic DNA was prepared from a loop-full of bacteria per the manufacturer's directions (Qiagen DNeasy Blood & Tissue Kit). Briefly, harvested bacteria were resuspended in enzymatic lysis buffer and incubated at 37°C for 30 minutes. Proteinase K and buffer AL were added to the cell suspension which was subsequently incubated at 56°C for an additional 30 minutes. After the addition of ethanol, the mixture was applied to a DNeasy Mini spin column. Following centrifugation and two wash steps, DNA was eluted from the column with Buffer AE.

### Genome sequencing, assembly and annotation

Newbler [2.0.1-PreRelease-3/30/2009], (Roche), was used to assemble the PNI0373 genome. Default Newbler parameters were used along with a “-consed” option to produce a full consed output and a “-rip” option, which assures that each read is placed in only one contig. Both of these options were used to generate an assembly format, which could be improved by manual efforts. The generated draft assembly was then finished to a high quality standard using targeted PCR and Sanger sequencing to resolve ambiguous bases, correct misassembled regions and fill gaps.

Genome annotation was performed using a pipeline developed as part of the NIH Human Microbiome Project at the Genome Institute at Washington University. Non-coding RNA genes were identified using tRNAscan-SE [1.23], Infernal 1.0/Rfam and RNAmmer [27]–[29]. GeneMark and Glimmer3 were used to predict protein-coding genes (CDSs) [30], [31]. CDS predictions were then processed through a gene selection pipeline, choosing a single representative from the multiple gene predictions in a region, based on a hierarchy of criteria. The resulting CDS set was analysed with psort-b, KEGG and Interpro-Scan to find functional domains and to assign gene ontology (GO) identifications and enzyme categorizations [32]–[35].

### Comparative analyses of complete pneumococcal genomes

Genomic data files for 11 complete pneumococcal genomes (Table 1) were obtained from the NCBI ftp site (ftp://ftp.ncbi.nih.gov/genomes/Bacteria/) in November 2009. Whole genome and proteome analyses were performed locally using the BLAST suite of programs as well as OrthoMCL for orthologous clustering [36]. Lineage-specific CDS were identified for each genome using the procedure from Lefébure *et. al*. 2007 [37] that defined taxa specific CDS as those not clustering during orthologous analysis, and which were at least 50 amino acids long with no significant BLASTP hit (evalue: 1e^−10^). Whole-genome and orthologous cluster alignments were generated using Mummer and MUSCLE, respectively [38]–[40]. A Mummer-based pipeline utilizing pairwise whole-genome alignments was used to produce dot plots and identify insertions, deletions and single nucleotide polymorphisms. Core orthologous clusters identified by OrthoMCL were organized into syntenic blocks using OrthoCluster [41]. Alien Hunter and IslandViewer were used to screen pneumococcal genomes for “atypical” sequence content indicative of horizontal gene transfer [42], [43]. Trees derived from presence or absence of genes as well as concatenated gene sequences were created using either Phylip or BioNJ and visualized with TreeIllustrator [44]–[46]. The circular representation of PNI0373 was produced with Circos [47]. The linear map of the full-length prophage present in PNI0373 was drawn using Genogator (http://www.kato.mvc.mcc.ac.uk/genogator/).

## Results and Discussion

### Post-invasive virulence of PNI0373 in murine models

**Figure 1:**
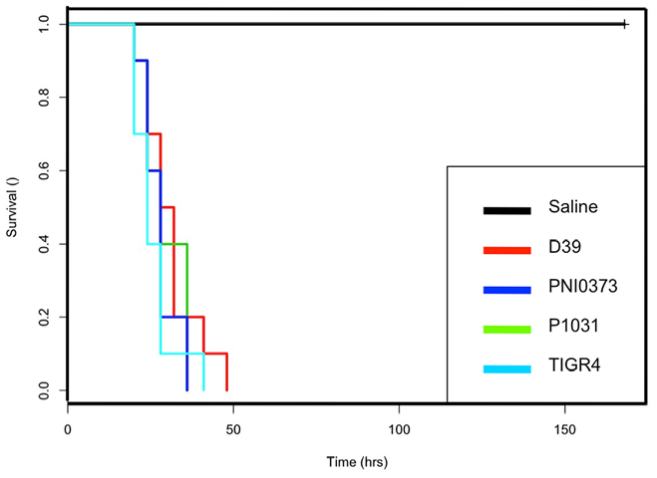
Murine survival following intraperitoneal challenge. Kaplan-Meier survival curves of groups of six female CD-1 mice after intraperitoneal challenge with TIGR4 (**— (light blue)**), D39 (**— (red)**), P1031(**— (green)**), PNI0373 (**—(dark blue)**) and R6/saline alone (**— (black)**).

Virulence is a multi-factorial process with microbial, environmental and host factors all playing a role in disease development and progression. Both clinical and animal studies demonstrate variability in serotype 1 virulence [48]–[50]. In order to assess the virulence of PNI0373 and P1031, two African clinical serotype 1 isolates, we employed a well-established model of post-invasive virulence in a murine model [51]. Following intraperitoneal challenge, mice receiving PNI0373, P1031, TIGR4 or D39 succumb to lethal infection within a 50 hour time period (Figure 1). No mortality was observed in mice receiving non-encapsulated R6, known for its avirulence in murine models [52]. The associated mortality for TIGR4 and D39 was consistent with previous reports at the same dosage level [50], [53]. High levels of bacteremia (>10^6^ cfu per 1 mL of blood) were recorded in mice challenged with PNI0373 within 8–10 hours post-infection and increased steadily until the mice became moribund (data not shown). Log-fold dilutions greater than 10^0^ cfu were lethal in all mice tested. At an approximate challenge dose of 10^0^ cfu, only 20% of the mice survived infection and showed no visible signs of illness seven days post-infection. Thus the PNI0373 isolate used for sequencing is highly virulent.

### PNI0373 general genomic features

**Figure 2:**
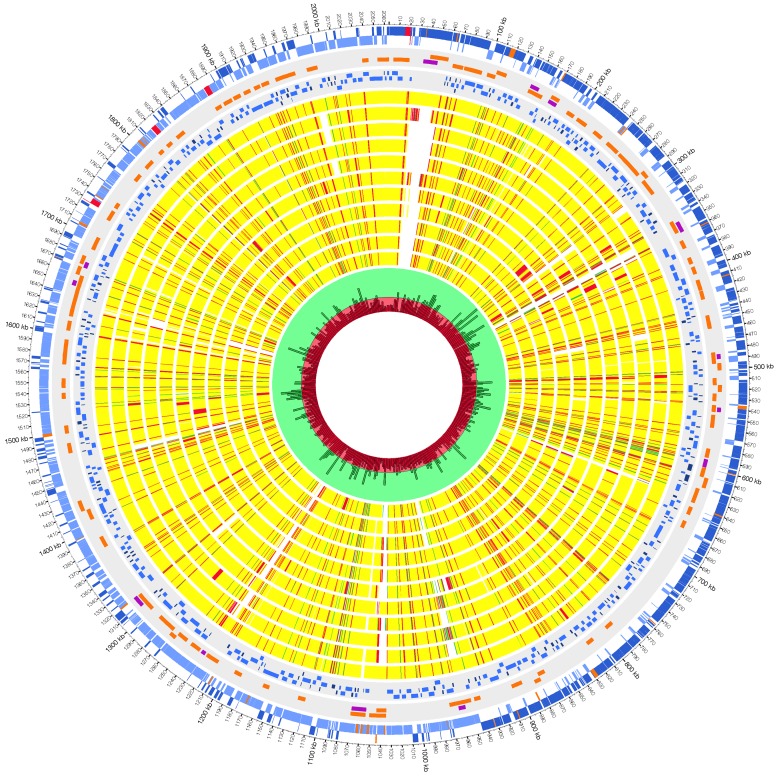
Graphical representation of the chromosome of *S. pneumoniae* PNI0373. Starting at the outermost circle moving to innermost, the circles display the following features. Circles 1 and 2 show forward and reverse strand genes, respectively (blue: coding, orange: pseudogenes, red: structural RNAs). Genomic regions displaying evidence of horizontal gene transfer are shown in circle 3 (orange: AlienHunter and purple: Islandviewer including SIGI-HMM, IslandPick and IslandPath_DIMOB). The fourth circle outlines the location and organization of pneumococcal core genes (dark blue: core gene singletons and light blue: syntenic blocks of 2 or more core genes. Circles 5 through 15 are BLASTN comparisons of other complete pneumococcal genomes to PNI0373 in following order: P1031, 70585, CGSP14, D39, G54, Hungary 19A-6, JJA, ATCC 700669, R6, Taiwan 19F-14 and TIGR4 with yellow showing high similarity (95–100%), red for intermediate (85–94%) and green for low (75–85%). The innermost circle displays the density per 5000 bp of the cumulative SNPs detected by comparison of 11 pneumococcal genomes to in PNI0373 (red shaded area: SNP density of 115 SNP per 5000 bp or less and green shaded area: SNP density between 115 and 345 SNPs per 5000 bp).

The PNI0373 genome was found to be a single circular chromosome consisting of 2,064,154 base pairs (bp) with a protein-coding capacity of 85.4% and a G+C content of 39.81%, comparable to pneumococcal genome averages (Figure 2). Gene prediction identified 2226 genes with an average length of 831 bp. Of these genes, 2117 are protein coding, 34 are pseudogenes and 75 are structural RNAs. Twelve rRNA genes are organized into four operons with the typical rRNA gene order of 16S, 23S, and 5S rRNA. A total of fifty seven tRNA genes, one less than the pneumococcal average, were predicted with cognates present for all amino acids. An additional six RNA genes were also identified by database comparison. A COG/KEGG function was assigned to 60.7% of the protein coding CDS. Over 99% of the predicted protein-coding sequences were found in at least one other pneumococcal genomes. The summarized genomic features of PNI0373 are shown in Table 1.

### Comparison to other pneumococcal genomes

**Figure 3:**
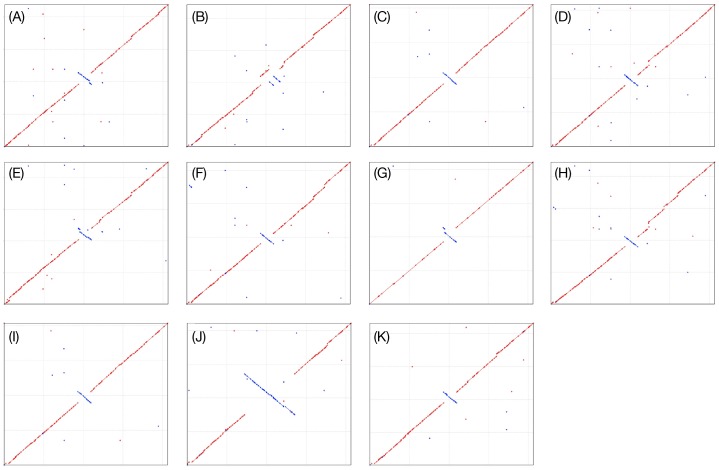
Comparisons of complete pneumococcal genomes to the PNI0373 reference. The eleven fully sequenced pneumococcal genomes were plotted against the PNI0373 genome. Relative to reference genome (PNI0373), the pneumococcal genomes are largely co-linear with minimal rearrangements. As noted, there is an inversion in all compared genomes ranging in size in the region surrounding the terminus of replication, which is often involved in structural variation. PNI0373 was plotted along the x-axis while the query genomes were plotted along the y-axis: (A) 70585; (B) CGSP14; (C) D39; (D) G54; (E) Hungary 19A-6; (F) JJA; (G) P1031; (H) ATTC 700669; (I) R6; (J) Taiwan 19F-14; and (K) TIGR4. Red line: conserved orientation; blue line: inverted orientation.

**Figure 4:**
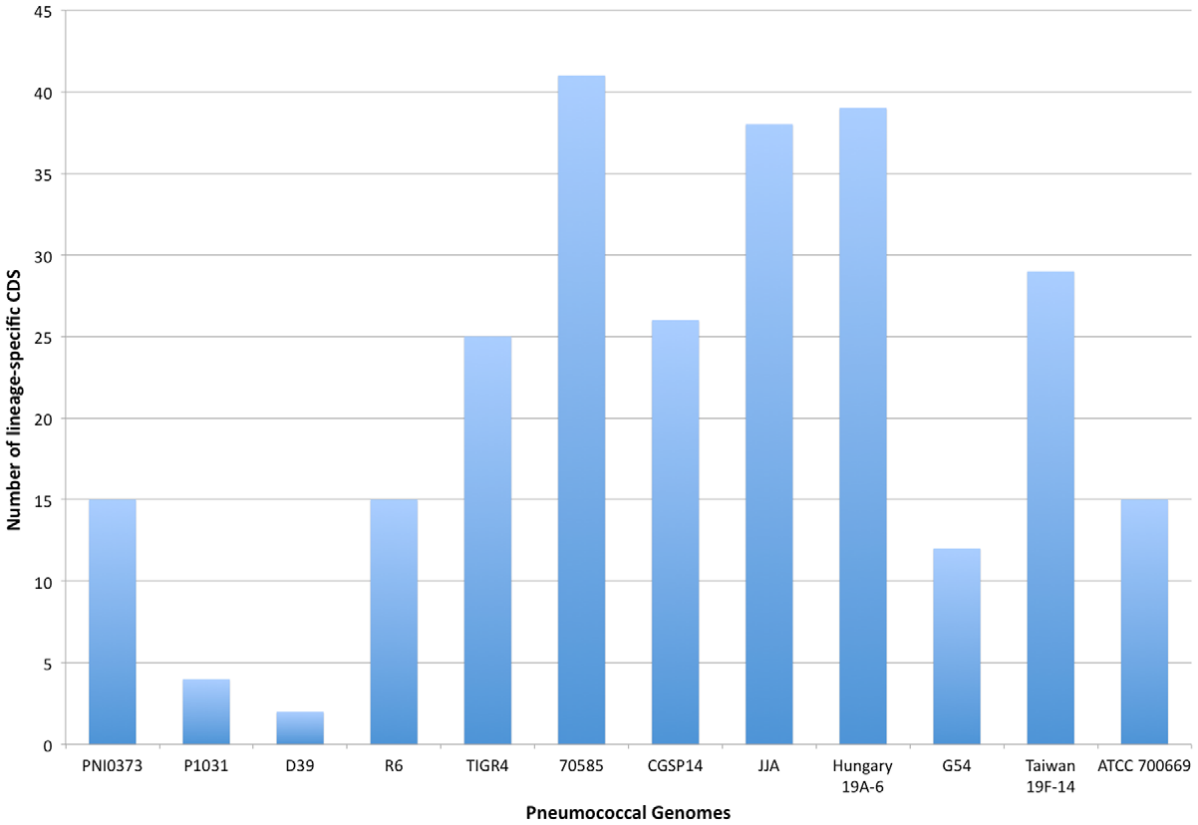
Lineage-specific CDS present in complete pneumococcal genomes. Genes present in only a single pneumococcal genome were identified using the following criteria: ≥50 codons in length and no significant blastp hits (<1e^−10^). Clonally related genomes, PNI0373 and P1031 as well as D39 and R6, contribute the least genic diversity to the pneumococcal pan-genome as they contain the fewest lineage-specific genes.

The PNI0373 genome is largely co-linear with other pneumococcal genomes, except for a ∼100 kb inversion surrounding the terminus of replication, as evidenced by a shift in GC skew (Figure 3). Orthologous clustering of 25174 CDS from the 12 complete pneumococcal predicted proteomes, including that of PNI0373, produced 2621 clusters. Only 796 CDS, 3% of the pan-pneumococcal proteome, did not cluster during orthologous analysis. PNI0373 contributed 15 lineage-specific CDS to the pneumococcal pan-genome (Figure 4). Together, the serotype 1 genomes contained only 19 lineage-specific genes similar to the number of lineage-specific CDS found in D39 and its clonal derivate R6. Other serotype pairs, CGSP14 and JJA as well as G54 and Taiwan 19F-14 contributed much more gene diversity to the pan-genome (Figure 4).

We identified 1441 clusters as comprising the pneumococcal core genome, representing 55% of all orthologous clusters. The addition of PNI0373 to the analysis of orthologs removed 15 orthologous clusters from the predicted core genome. Several of these clusters contain virulence-associated proteins as well as proteins possessing either transmembrane helices or the cell wall anchor LPXTG-motif (below). The majority of the core clusters are organized into syntenic blocks ranging in size from two to seventeen CDS, the median being three CDS per block.

**Figure 5:**
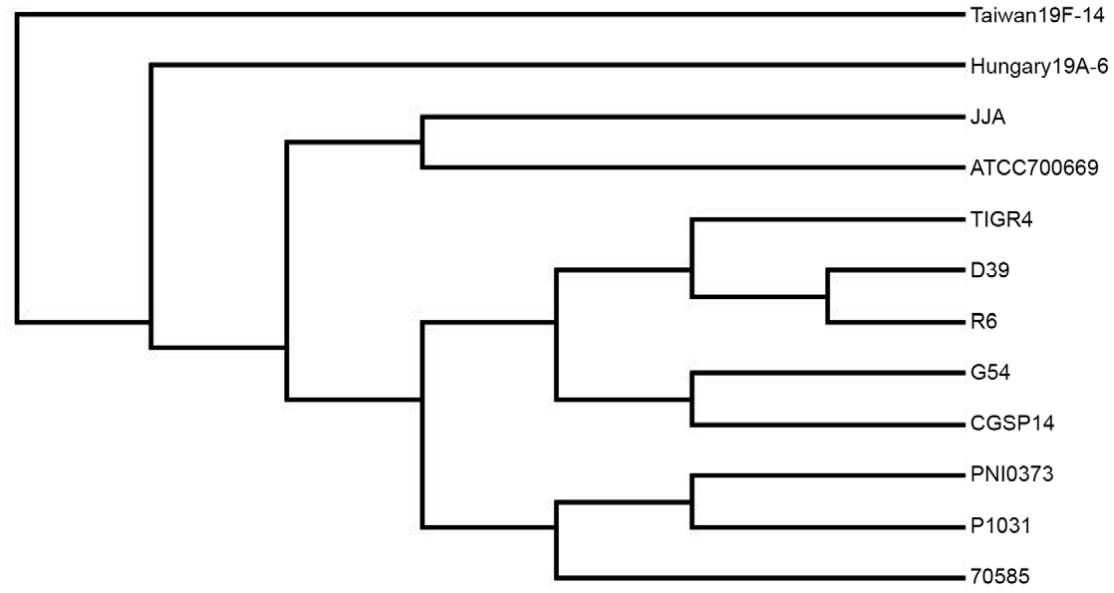
Gene content-based dendrogram of complete pneumococcal genomes. The presence or absence of orthologs within clusters for each genome was ascertained. After normalizing the data using the weighted average genome size from our data set, the above dendrogram was generated from our normalized gene content matrix in BioNJ. The two serotype 1 isolates, PNI0373 and P1031, clustered closely together indicating a strong relationship based on gene content. The serotype 5 genome,70585, also clustered closely with the serotype 1 genomes. Both serotype 1 and 5 pneumococci are rarely found in carriage and highly invasive. The dotted-line box highlights relationship of serotype 1 and serotype 5 genomes to each other.

Trees generated based on the presence or absence of orthologs comprising the full complement of orthologous clusters and the non-core fraction showed a close relationship between the two serotype 1 genomes as well as a close relationship between the serotype 1 isolates and 70585, a serotype 5 isolate (Figure 5). The pairs of serotype 14 and serotype 19F isolates, CGSP14/JJA and G54/Taiwan 19F-14 respectively, showed a much more distant relationship to each other highlighting the intraserotype homogeneity seen in the serotype 1 genomes. Pairwise comparison of orthologs between all 12 genomes further demonstrated a significant similarity between the serotype 1 genomes (Table 2). PNI0373 and P1031 shared 1888 orthologs and differed by 237, both three standard deviations from the mean (shared: 1753±38 S.D. and difference: 449±67 S.D.). The number of orthologs shared between PNI0373 and 70585 was also two standard deviations above the mean. Only the D39 and R6 pair possessed fewer differences in total orthologs present in their genomes, an expected result given the relationship of these strains.

**Table 2:** Pairwise comparisons of orthologous content.

	70585	CGSP14	D39	G54	Hungary 19A-6	JJA	P1031	PNI0373	ATCC 700669	R6	Taiwan 19F-14	TIGR4
70585		1799	1744	1799	1799	1817	1806	1838	1747	1766	1758	1764
CGSP14	461		1720	1806	1755	1777	1745	1757	1782	1754	1730	1774
D39	418	475		1726	1674	1729	1711	1743	1696	1812	1698	1723
G54	392	387	394		1777	1765	1774	1784	1745	1749	1741	1726
Hungary 19A-6	462	559	568	446		1760	1769	1743	1782	1673	1767	1727
JJA	390	479	422	434	514		1765	1777	1803	1750	1751	1763
P1031	409	540	455	413	493	465		1888	1738	1721	1754	1702
PNI0373	366	537	412	414	566	462	237		1726	1776	1750	1731
ATCC 700669	498	437	456	442	438	360	487	532		1711	1698	1731
R6	435	468	199	409	631	441	496	407	487		1707	1741
Taiwan 19F-14	436	501	412	410	428	424	415	444	498	455		1736
TIGR4	447	436	385	463	531	423	542	505	455	410	405	

Comparing each genome to the other 11 genomes in our analysis, we calculated the number of shared and differing orthologs for each pair. The total number of differing orthologs equaled the sum of subtracting the number of shared orthologs from the total orthologs for each genome. We then determined the average shared and differing ortholog count along with standard deviations from the mean, 1754±38 and 499±67. The upper diagonal contains shared ortholog values while the lower diagonal shows differing ortholog values. Shared or differing ortholog counts that were two standard deviations from mean are highlighted in blue while those three standard deviations from the mean are in gold.

### Conserved regions and SNPs unique to serotype 1 isolates

We identified three genomic regions that were conserved in the two serotype 1 genomes and either absent or highly divergent in other pneumococcal genomes. All three regions exhibit evidence of horizontal gene transfer and encode proteins with potential roles in virulence (Figure 2).

**Figure 6:**
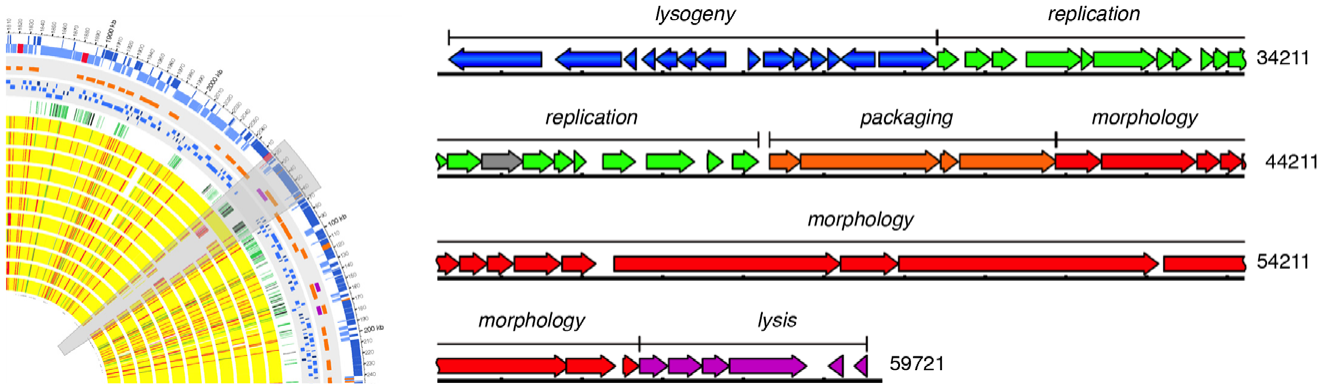
Genomic location and structure of prophage in PNI0373. The inset depicts the genomic location of the identified prophage, φPNI0373, and highlights other features in the region across the genomes analyzed. The predicted modular structure of φPNI0373 is shown with the gene content and orientation for each unit indicated.

The first region (coordinate 24000 bp) contains an integrated 35.5 kb bacteriophage, φPNI0373, predicted to encode 58 genes, with a G+C content of 40.1%, similar to the average G+C content of PNI0373 (Figure 6). This prophage is found integrated between HMPREF1038_00023 and HMPREF1038_00084. Orthologs of HMPREF1038_00023 and HMPREF1038_00084 are found flanking prophages within several other pneumococcal genomes including SP3-BS71, SP11-BS70, SP14-BS69, Hungary 19A-6, CDC3059-06, and CDC1873-00. We were able to identify the previously shown attachment core sequence (5′-CTTTTTCATAATAATCTCCCT-3′) for φSP3-BS71 adjacent to the integrase and endolysin of φPNI0373 [54]. This sequence is also found at two other sites in the PNI0373 genome: between HMPREF1038_00314 and HMPREF1038_00315 and between HMPREF1038_00316 and HMPREF1038_00317. Based on sequence and protein similarity, prophages integrated between orthologs of HMPREF1038_00023 and HMPREF1038_00084 cluster together forming a closely related group separate from other pneumococcal temperate prophages [54]. Of the previously studied pneumococcal prophages in this group, φPNI0373 is most closely related to φSP3-BS71, sharing 71% identity. Interestingly, φPNI0373 showed 99.97% identity with φP1031, located in the same region in P1031, further emphasizing the extent of genetic similarity between these genomes.

φPNI0373 has the characteristic conserved organization seen in other temperate pneumococcal phages (Figure 6). Its genome is arranged into five modular units controlling lysogeny, replication, packaging, morphology and lysis [55]. Within the lysogeny module, there are two hypothetical proteins of unknown function unique to φPNI0373 and φP1031. Downstream in the morphology module, HMPREF1038_00074 has sequence similarity to the *Streptococcus mitis* SM1 phage-encoded PblB protein. In *S. mitis*, PblB functions together with PblA to mediate bacterial adhesion to human platelets and thus directly contributes to the pathogenesis of infective endocarditis [56]. φPNI0373 does not encode a protein with sequence similarity to PblA. Several of the related temperate pneumococcal phages also encoded *pblB*-like genes with or without a *pblA*-like gene. These *pblB*-like genes may participate in bacterial adhesion and thus contribute to virulence although the mechanism by which this occurs may differ slightly from that seen in *S. mitis* given that in some cases PblA is absent. Furthermore, given the presence of a *pblB*-like gene in serotypes with lower invasive ratios (i.e. serotype 6A, 11 and 14), this phage-encoded protein does not appear to be solely responsible for the highly invasive phenotype of serotype 1 isolates, though it may contribute to virulence in some manner. Additionally following the endolysin, there are an additional two conserved hypothetical proteins of unknown function prior to encountering the flanking attachment sequence.

The capsular polysaccharide biosynthesis locus of PNI0373 shares 99% sequence identity with the serotype 1 capsular locus in P1031 and 519/43 [57]. We detected 1227 positions (1215 SNPs and 12 indels) within the core sequence in which serotype 1 genomes differed from that of the other serotypes (Table S2). Approximately 179 positions (170 SNPs and 9 indels) were located within intergenic regions with the remainder located within 369 CDS. Fifty-one percent of the SNPs within coding sequences resulted in synonymous changes at the protein level while the three coding indels resulted in frameshifts. Examination of the coding sequences possessing these serotype 1-specific variants revealed the presence of 72 previously-identified virulence-associated genes (Table S2).

### Virulence factors and PNI0373

#### Competence proteins

Several PNI0373 genes involved in competence regulation pathways are absent or contain deleterious mutations in PNI0373. The competence locus, ComCDE, encodes three proteins, which act in concert to regulate transcriptional activitation of genes involved in competence. While the histidine kinase receptor, ComD, and competence-stimulating peptide, ComC are present, there is no evidence of any open-reading frame corresponding to competence protein E (comE), the cognate response regulator of the comD. There is a non-open reading frame DNA fragment, 198 bp in length upstream of ComD, which corresponds to the N-terminal 66 amino acids of ComE. The receiver region of signal transduction response regulators is typically located at the N-terminus of TCS response regulators and this 198 bp fragment does not contain this domain in its entirety.

The late competence gene *coiA*, acting downstream of ComE contains a single base insertion at position 617 resulting in a frameshift and the introduction of multiple early termination codons. Following DNA uptake, CoiA is required for genetic transformation. The CoiA frameshift and ComE deletion in combination suggest that PNI0373 no longer remains able to be naturally transformable. Several attempts to transform PNI0373 with naked DNA failed to demonstrate DNA uptake by this isolate (data not shown).

#### Choline-binding proteins

Pneumococci contain several choline-binding proteins (CBPs) possessing highly conserved choline-binding domains (CBDs) of 20 amino acid direct repeats which bind phosphorylcholine in the cell wall. Approximately 16 CBPs have been identified to date with nine implicated in virulence either through adhesion or enzymatic activity [58]–[61]. The genes for six of these nine are intact within the PNI0373 genome (*pspA*, *pce*, *lytA*, *lytB*, *lytC*, and *pspC*), while *pcpA* and *cbpF* are absent (despite their proven role in virulence) and *cbpG* is truncated [60], [62].

#### Colonization-associated factors

Several pneumococcal proteins have been identified as promoting pneumococcal colonization within the nasopharyngeal niche. Given the limited amount of serotype 1 carriage acquisitions, we searched the genome of PNI0373 for the presence of these colonization-associated genes. Three colonization-associated genes, *strH*, *eno*, and *nanA* are absent from PNI0373 while four others, *hyl*, *cbpA*, *pavA*, and *bgaA*, are present.

#### Other virulence factors

A number of studies have examined the contribution of individual pneumococcal genes to bacterial pathogenesis in various genetic backgrounds including TIGR4, G54 and R6 [60], [61], [63]. A total of 319 pneumococcal genes have been associated with virulence via knockout attenuation studies in animal models. We surveyed the predicted proteome of PNI0373 and found that 90% of identified virulence-contributing pneumococcal genes were present in the genome (see Table S1). Furthermore, three-fourths of the virulence factors present in PNI0373 were determined to be part of the core pneumococcal genome. Thirty-eight of the identified virulence-associated genes were absent from PNI0373, with all but three of these also absent in P1031.

Neither of the previously identified pilus operons, PI-1 or PI-2, was present in PNI0373. The absence of PI-1 was expected as this operon does have an association with highly invasive isolates, such as serotype 1 strains [64]. PI-2, originally detected within a serotype 1 strain (INV104B) and confirmed to be present in other serotype 1 isolates, was notably absent from PNI0373 as well as P1031 [65]. The flanking genes of *pepT* and *hemH* to P1–2 are present in PNI0373. The putative insertion site for PI-2 located between these two genes (5′-TCCTTTT-3′) contains a single base substitution at the sixth base position (T:G) in PNI0373 [65]. Additionally, the non-hemolyitc allele of pneumolysin previously linked with dominant clones of outbreak-prone serotypes, 1 and 8, was not present in either PNI0373 or P1031 [66], [67].

### Protein-based vaccine candidates

Antibody to several pneumococcal proteins has been shown to protect experimental animals against pneumococcal challenge and, in preliminary vaccine trials, perhaps to be protective in humans. From literature searches, we identified 29 pneumococcal proteins demonstrating protective efficacy in animal models [68], [69]. Twenty-five of these 29 were present within PNI0373. All of the most promising protein candidates, particularly those having undergone various stages of preliminary clinical testing, are present and highly conserved [70]. Thus, those vaccine candidates are likely to have similar protective efficacy against serotype 1 isolates closely related to PNI0373 and P1031.

### Accessory regions

We also surveyed the PNI0373 genome to determine the presence or absence of the 41 accessory regions or regions of diversity (AR), so-called because of their differential distribution pattern in various pneumococcal genomes [71]–[73]. Seventeen AR were intact within the PNI0373 genome with an additional 12 AR partially present (Table S3). Altogether, PNI0373 contained 13 of the 24 accessory regions known to have an association with virulence. An additional four virulence-associated AR were partially present in PNI0373, but these partial sequences did not encode the genes contributing to virulence for these regions. The pattern of AR distribution in PNI0373 and P1031 was very similar to that seen in other serotype isolates belonging to the same clonal complex [71].

## Conclusions

*S. pneumoniae* serotype 1 is a highly invasive serotype responsible for a significant proportion of pneumococcal disease within Africa and Asia. Despite its unique epidemiological and clinical features, the genome of *S. pneumoniae* serotype 1 strain PNI0373 shared a large degree of similarity with genomes from other serotypes. Most previously identified virulence-associated genes were present within PNI0373 as well as part of the core genome, indicating perhaps a commonality for all pneumococci in their mechanisms of pathogenesis.

Furthermore, comparative analysis revealed a high degree of similarity between the two serotype 1 isolates, more so than seen with other intra-serotype comparisons, confirming MLST-based observations on clonality of serotype 1 [74], [75]. Neither of the sequenced serotype 1 isolates added much diversity to the pneumococcal pan-genome due to the relative low number of lineage-specific coding sequences in each. Given the high burden of serotype 1 pneumococcal disease in developing countries, inclusion of serotype 1 antigens within either capsule- or protein-based vaccines would have tremendous potential to control this pathogen. From our analysis, the majority of the potential well-studied pneumococcal protein candidates are present within both serotype 1 genomes and well conserved providing some suggestion as to the coverage such vaccines would possess.

## Supporting Information

Table S1 **Detection of Virulence Genes.** An exhaustive literature search revealed 313 pneumococcal proteins shown to contribute to virulence in animal models and culture assays. The presence of each of the virulence-associated proteins was determined for each of the 12 fully sequenced *S. pneumoniae* genomes. A “+” indicates the gene is present while a “−” indicate its absence. The core column indicates genes that were known to be part of the pneumococcal core genome prior to the introduction of PNI0373 and/or P1031 into the analysis.
(XLS)

Table S2 **Variant Detection in Core Pneumococcal Sequence – Serotype 1-specific positions.** The non-repetitive, unique core sequence for the 12 complete pneumococcal genomes analyzed was queried for presence of variants. In particular, we searched for variants in which serotype 1 genomes, PNI0373 and P1031, shared alleles while genomes from other serotypes differed. (sSNP: synonymous SNP; nsSNP-c: conservative non-synonymous SNP; nsSNP-nc: non-conservative non-synonymous SNP; nsSNP-ns: nonsense non-synonymous SNP; nsSNP-rt: read-through non-synonymous SNP indicating mutation in stop codon).
(XLS)

Table S3 **Accessory regions in PNI0373.** The presence or absence of previously identified pneumococcal regions of diversity/accessory regions was assessed for both PNI0373 and P1031 [71]–[75]. The presence or absence of accessory regions was determined using BLASTN and BLASTP searches of AR sequences and genes encoded within AR. The criteria to define AR as present (+), partial (+/−) or absent (−) is as follows: (a) present (+) if greater than 85% of region sequence and/or genes encoded in region were present in a conserved/syntenic block; (b) partial (+/−) if between 25–85% of region sequence and/or encoded genes in block present; anc (c) absent (−) if less than 25% of region sequence and/or encoded genes present.
(XLS)
